# Profiles in Cytotoxicity: A First Step Toward Chemical-Specific Adjustment Factors

**DOI:** 10.1289/ehp.123-A136

**Published:** 2015-05-01

**Authors:** Julia R. Barrett

**Affiliations:** Julia R. Barrett, MS, ELS, a Madison, WI–based science writer and editor, is a member of the National Association of Science Writers and the Board of Editors in the Life Sciences.

Human risk assessment of chemicals has traditionally relied on expensive and time-consuming methods to determine toxicity and set regulatory limits for exposure. High-throughput cell-based screening assays are a practical option for rapidly assessing individual chemicals and establishing reliable safety limits.^1^^,^^2^^,^^3^ A new study in *EHP* demonstrates that these assays can also be used to estimate the range of responses within human populations.^4^

Traditional risk assessment is based on identifying the amount of a chemical that causes no observable effect in exposed animals. To extrapolate the results to human health, that amount is divided by an interspecies uncertainty factor. The result is divided by an additional safety factor of 10 to account for individuals who might be particularly susceptible to harm.

**Figure:**
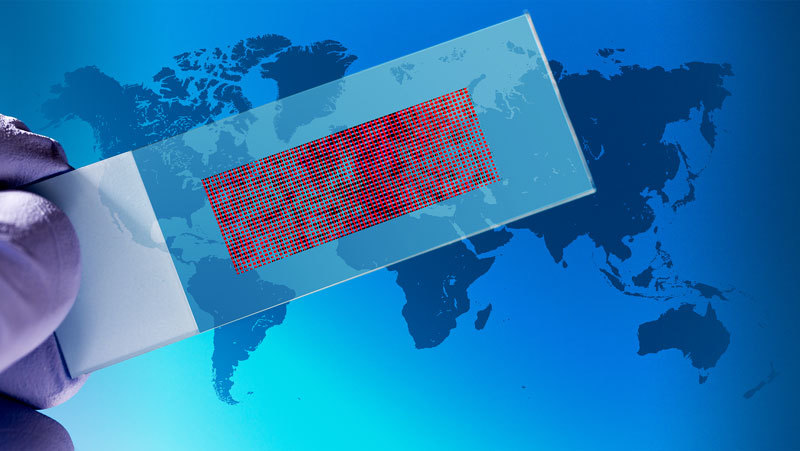
The 1000 Genomes Project is on track to establish more than 3,000 immortalized lymphoblastoid cell lines from blood samples provided by individuals from 25 distinct populations around the world. © Science Photo/Shutterstock, Alexandra Dreval/Shutterstock

Risk estimates still have a fair amount of uncertainty, however, and the traditional risk assessment approach is too cumbersome to effectively evaluate the enormous and growing backlog of untested chemicals.^1^ These challenges have driven the development of efficient *in vitro* testing methods and initiatives to screen thousands of chemicals and to determine their biological effects and the exposure levels at which they occur.^3^ What these initiatives generally lack is an accounting for interindividual variability—a gap the current study aimed to fill.^4^

“Even though there are hundreds of cell-based and cell-free models, and many chemicals are being sent through them, we really are not addressing the key point that’s important for regulatory decision making, which is how much interindividual variability potentially exists in these responses,” says coauthor Ivan Rusyn, a professor of veterinary integrative biosciences at Texas A&M University.

To accomplish their goal, the researchers used a high-throughput screening assay that can rapidly assess cytotoxicity (the ability of a compound to kill cells). The authors used cells derived from the 1000 Genomes Project,^5^ an international collaboration that is on track to establish more than 3,000 immortalized lymphoblastoid cell lines from blood samples provided by individuals from 25 distinct populations around the world.^6^ These cell lines and their mapped genomes are available to researchers worldwide for studying genetic contributions to disease—or, as in this case, response to toxic insult.

The researchers used 1,086 cell lines—essentially the equivalent of 1,086 individuals representing a multinational population—to assess the cytotoxicity of 179 chemicals at 8 different concentrations. They analyzed the data with an eye toward variations in responses between lines. “Some chemicals showed no effect over the range investigated, while others were highly toxic; these results can help place higher research priority on the more toxic compounds,” says coauthor Fred Wright, a professor of statistics and biological sciences at North Carolina State University.

The researchers also compared genetic variation in the cell lines against observed toxicity. Interestingly, this analysis spotlighted genes for specific membrane proteins and solute carrier transporters, with a particular focus on *SLC7A11*. One variant of this gene was associated with susceptibility variation for several chemicals; the gene itself has previously been linked with chemotherapeutic drug resistance.^7^

Barbara Wetmore, a senior research investigator at The Hamner Institutes for Health Sciences, characterizes the study as an important step in increasing the understanding of how much genetic variability contributes to the overall variability within a population. “It’s just been one of these black holes,” says Wetmore, who was not involved in the study. “We know we need to be concerned with variability, and we want to be sure to protect across all populations, but it’s something that, until recently, we haven’t had appropriate tools to apply.”

Wetmore says the work and findings of the current study will be extremely important in guiding future efforts to address other contributors to population variability (e.g., pharmacokinetic variability, target organ susceptibilities) for which high-throughput tools do not yet exist. Furthermore, she adds, advances like this will contribute greatly to our ability to ensure adequate protection across all sensitive populations and life-stages.^8^

This study accomplished its primary goal of confirming that *in vitro* cytotoxicity testing can be used to gather information on the range of interindividual differences in response to toxicants. In addition, the data recently proved useful in supporting computer-based toxicity predictions based on chemical structures and an individual’s genetic makeup.^9^ “We really are trying to provide the science to inform decisions and to get away from default assumptions that one size fits all for interspecies and interindividual variability,” says Rusyn. “Understanding the range of interindividual variability is a step towards reducing uncertainties in risk assessment.”
